# Supplementary material for: Assessing Healthcare Professionals’ Identification of Paediatric Dermatological Conditions in Darker Skin Tones
Source: Children (Basel). 2022 Nov 15;9(11):1749. doi: 10.3390/children9111749 (PMC9688675; doi:10.3390/children9111749)
Supplement: Supplementary file 1 [file children-09-01749-s001.zip › children-1996393-supplementary.pdf]

Online supplementary material:

Diagnosis	Total	Correct	Correct %
Cafe-au-lait macule	75	71	95%
Scrofuloderma	64	60	94%
Systemic lupus erythematosus	40	37	93%
Chicken pox scars	71	64	90%
Staphylococcal abscess	66	56	85%
Burn	283	239	84%
Pemphigus	64	54	84%
Ichthyosis	37	31	84%
Wart	166	139	84%
Chicken pox	43	35	81%
Hyperkeratosis factitia	56	45	80%
Dermatosis papulosis nigra	57	43	75%
Psoriasis	52	39	75%
Pyogenic granuloma	44	33	75%
Oral candidiasis	46	33	72%
Staphylococcal infection	58	41	71%
Vitiligo	48	33	69%
Tinea faciei	54	36	67%
Erythema nodosum	139	92	66%
Infected eczema	64	42	66%
Bullous impetigo	65	41	63%
Crusted scabies	48	30	63%
Chemical burn	70	43	61%
Meningococcal septicaemia	61	37	61%
Hand, foot and mouth disease	117	69	59%
Napkin dermatitis	69	40	58%
Intertrigo (due to Candida)	49	28	57%
Measles	52	27	52%
Lichen planus	60	30	50%
Nodular prurigo	6	3	50%
Henoch-Schonlein purpura	95	47	49%
Omphalitis	47	22	47%
Leukaemia cutis	54	25	46%
Urticaria	71	31	44%
Acne vulgaris	62	27	44%
Pityriasis versicolor	184	80	43%
Discoid lupus	56	23	41%
Epidermal naevus	59	24	41%
Eczema	205	83	40%
Herpes simplex virus	259	99	38%
Traumatic fissures	72	27	38%
Tinea corporis	106	39	37%
Toxic epidermal necrolysis	47	17	36%
Abscess	61	20	33%
Larva migrans	61	20	33%
Ecthyma	60	19	32%
Ecthyma gangrenosum	55	16	29%
Beckers naevus	62	17	27%
Scabies	249	67	27%
Conjunctivitis	65	13	20%
Chemotherapy	43	8	19%
Dog bite	41	7	17%
Kawasaki disease	40	6	15%
Miliaria crystallina	54	6	11%
Foliculitis	54	4	7%
Syphilis	34	2	6%

Figure S1 Percentages of correctness for all 56 diagnoses.

Table S1. Characteristics of participants answered eight or more questions correctly.

	Quiz score <8 (n=385)		Quiz score ≥ 8 (n=47)		p-value
	n	Col%	n	Col%	
**Continent**					0.605
Europe	223	58%	29	62%	
Oceania	87	23%	11	23%	
America	33	9%	3	6%	
Asia	28	7%	1	2%	
Africa	9	2%	2	4%	
Latin America	5	1%	1	2%	
**Ethnicity**					0.581
White	266	69%	37	79%	
Asian/Oriental	84	22%	6	13%	
Black or African	14	4%	2	4%	
Hispanic or Latino	6	2%	0	0%	
Unclassified	15	4%	2	4%	
**Majority Training Resources**					0.938
White skin	283	74%	36	77%	
A mix of skin tones	95	25%	11	23%	
Darker skin tones	7	2%	0	0%	
**Profession**					0.704
Medic	284	74%	35	74%	
Nursing	40	10%	6	13%	
Advanced nursing practitioner	35	9%	2	4%	
Paramedic & Other HCP	10	3%	1	2%	
Other*	16	4%	3	6%	
**Specialty**					0.016
Paediatrics	162	42%	16	34%	
Emergency medicine	83	22%	7	15%	
Primary care	50	13%	10	21%	
Emergency paediatrics	41	11%	5	11%	
Dermatology	8	2%[a]	5	11%[b]	
Other†	41	11%	4	9%	
**Experience**					0.463
Student	33	9%	1	2%	
1-2 years	39	10%	5	11%	
3-5 years	86	22%	12	26%	
6-10 years	125	32%	19	40%	
11 years +	102	26%	10	21%	
**Confidence in diagnoses**					0.300
Generally uncertain if correct	166	43%	15	32%	
Sometimes uncertain but clinically safe	200	52%	30	64%	
Confident across range of skin tones	19	5%	2	4%	

** Including medical doctors/primary care practitioner (all grades) and medical student (n=1), physician associate or assistant (3). † Including paramedic (7) and clinical pharmacist (1), pharmacist (1), physiotherapist (1), podiatrist (1)*

Values in the same row not sharing the same subscript are significantly different at p<0 .05 in the two-sided test of equality for column proportions.
